# Advanced glycation end products impair bone marrow mesenchymal stem cells osteogenesis in periodontitis with diabetes via FTO-mediated *N*^6^-methyladenosine modification of sclerostin

**DOI:** 10.1186/s12967-023-04630-5

**Published:** 2023-11-04

**Authors:** Jie Zhou, Yanlin Zhu, Dongqing Ai, Mengjiao Zhou, Han Li, Guangyue Li, Leilei Zheng, Jinlin Song

**Affiliations:** 1 College of Stomatology, Chongqing Medical University, Chongqing, People’s Republic of China; 2 Chongqing Key Laboratory of Oral Diseases and Biomedical Sciences, Chongqing, China; 3 Chongqing Municipal Key Laboratory of Oral Biomedical Engineering of Higher Education, Chongqing, China

**Keywords:** Diabetes mellitus, Periodontitis, Bone marrow mesenchymal stem cells, Osteogenesis, Advanced glycation end products, *N*^6^-methyladenosine

## Abstract

**Background:**

Diabetes mellitus (DM) and periodontitis are two prevalent diseases with mutual influence. Accumulation of advanced glycation end products (AGEs) in hyperglycemia may impair cell function and worsen periodontal conditions. *N*^6^-methyladenosine (m^6^A) is an important post-transcriptional modification in RNAs that regulates cell fate determinant and progression of diseases. However, whether m^6^A methylation participates in the process of periodontitis with diabetes is unclear. Thus, we aimed to investigate the effects of AGEs on bone marrow mesenchymal stem cells (BMSCs), elucidate the m^6^A modification mechanism in diabetes-associated periodontitis.

**Methods:**

Periodontitis with diabetes were established by high-fat diet/streptozotocin injection and silk ligation. M^6^A modifications in alveolar bone were demonstrated by RNA immunoprecipitation sequence. BMSCs treated with AGEs, fat mass and obesity associated (FTO) protein knockdown and sclerostin (SOST) interference were evaluated by quantitative polymerase chain reaction, western blot, immunofluorescence, alkaline phosphatase and Alizarin red S staining.

**Results:**

Diabetes damaged alveolar bone regeneration was validated in vivo. In vitro experiments showed AGEs inhibited BMSCs osteogenesis and influenced the FTO expression and m^6^A level in total RNA. FTO knockdown increased the m^6^A levels and reversed the AGE-induced inhibition of BMSCs differentiation. Mechanically, FTO regulated m^6^A modification on SOST transcripts, and AGEs affected the binding of FTO to SOST transcripts. FTO knockdown accelerated the degradation of SOST mRNA in presence of AGEs. Interference with SOST expression in AGE-treated BMSCs partially rescued the osteogenesis by activating Wnt Signaling.

**Conclusions:**

AGEs impaired BMSCs osteogenesis by regulating SOST in an m^6^A-dependent manner, presenting a promising method for bone regeneration treatment of periodontitis with diabetes.

**Supplementary Information:**

The online version contains supplementary material available at 10.1186/s12967-023-04630-5.

## Introduction

Diabetes mellitus (DM) is a metabolic disease with multiple causes. Defects in insulin production or function disrupt glucose and lipid metabolism, resulting in chronic hyperglycemia with vascular problems, skeletal system degeneration, delayed tissue healing and other complications [1]. Periodontitis is an infectious disease featured with periodontal tissue inflammation, alveolar bone resorption and tooth loss. The two diseases have overlapping effects. Diabetes is considered to be a risk factor for periodontitis, and the periodontal therapy could also reduce blood glucose level of patients [2]. It is widely accepted that diabetes could weaken the resistance to local adverse stimuli, exacerbate gingivitis, predispose to periodontal abscess, accelerate alveolar bone loss and damage tissue regeneration [3].

Given the abnormal metabolism, hyperglycemia alters local microbial composition, provokes immune dysfunction, induces inflammatory factors and accelerates the destruction of periodontal tissue [4]. Meanwhile, hyperglycemia inhibits the activity of osteoblasts and fibroblast, reduces the synthesis of collagen and bone matrix, which affect the reconstruction of periodontal tissue [5]. Hyperglycemia has been related to the formation of advanced glycation end products (AGEs), methylglyoxal (MGO), and other byproducts that impair cellular function as well as exacerbate oxidative stress and inflammation reactions [6]. Accumulative evidence suggests that AGEs inhibit the function of osteoblasts and chondrocytes, accelerate osteocyte senescence and apoptosis, alter bone turnover rate and reduce biomechanical strength [7, 8]. As a key source of periodontal tissue regeneration, bone marrow mesenchymal stem cells (BMSCs) play a crucial role in the process of alveolar bone remodeling. AGEs suppressed BMSC proliferation and osteogenesis while provoking apoptosis, resulting in a disturbance in alveolar bone homeostasis [9]. However, the explicit mechanisms under which the impaired osteogenesis of BMSCs induced by AGEs remain unclear.

*N*^6^-methyladenosine (m^6^A) is the common epigenetic modification in eukaryotic RNAs, and it participates in RNA processing, splicing, nuclear export, stability and translation efficiency, as well as being closely related to various biological processes [10]. M^6^A methylation is introduced into RNAs by the methyltransferase complex (MTC) consists of methyltransferase-like 3 (METTL3), methyltransferase-like 14 (METTL14) and Wilm tumor associated protein (WTAP), and is primarily erased by demethylase such as fat mass and obesity associated (FTO) protein and alkB homolog 5 (ALKBH5). Studies have confirmed that FTO levels were positively correlated with obesity, insulin resistance, blood glucose, the risk of complications, and FTO were up-regulated in vessels, liver and skeletal muscles of DM patients and animal models, indicating the FTO-m^6^A axis could be a potential therapeutic target for diabetic complications [11–13]. Recent studies revealed that m^6^A methylation regulated bone development and pathophysiology processes such as osteoporosis, osteoarthritis and osteosarcoma [14, 15]. On the cellular level, m^6^A modification has been shown to mediate BMSC fate commitment, osteoblast differentiation and osteoclast-induced bone absorption [16, 17]. GDF11-FTO-PPAR axis was reported to promote MSC differentiate into adipocytes and inhibit bone formation during osteoporosis [18]. These findings indicated that FTO-m^6^A axis may be involved in bone homeostasis in periodontitis with diabetes.

Therefore, the up-regulation of FTO in DM and its negative effect on osteogenesis may play a role in the progression and treatment of periodontitis with diabetes. In this study, we evaluated the effects of AGEs on BMSCs, elucidated the mechanism of m^6^A methylation modification, and provided a prospective therapeutic insight for periodontitis management in DM patients.

## Materials and methods

### Mice and type 2 diabetes mellitus (T2DM) establishment

The animal experiments were complied with the ARRIVE (Animal Research: Reporting of In Vivo Experiments) guidelines and approved by the Ethics Committee of the College of Stomatology, Chongqing Medical University. 6-week-old male C57BL/6 mice were purchased from the SJA laboratory animal CO. LTD (Hunan, China) and were housed in a specific pathogen-free condition: room temperature 22 ± 2 °C, 12 h light/dark cycle and water ad libitum. 36 mice were randomly divided into two groups (n = 18/group) and fed with standard diet or high fat/high glucose diet (HFHG, D12492, Research Diets, NJ, USA) respectively. After 8 weeks, HFHG-fed mice weighed up to 30–35 g and were intraperitoneally injected with streptozotocin (STZ, 35 mg/kg, Sigma-Aldrich, MO, USA) for 3 consecutive days, while mice on the standard diet were given citrate buffer. 7 days after the last injection and then once a week after that, blood glucose levels were measured. T2DM was diagnosed in mice with fasting blood glucose levels higher than 16.7 mM and clinical symptoms (increased food and water consumption, increased urine output and weight loss).

### Experimental periodontitis model

Periodontitis was induced in Control and T2DM groups as described previously after blood glucose had been stable for 2 weeks [19]. The primary result was the distance of cemento-enamel junction (CEJ)-alveolar bone crest (ABC). Significant intergroup differences of 0.25 mm (standard deviation [SD] = 0.05 mm) in control and 0.45 mm (SD = 0.2 mm) in periodontitis were determined on the basis of published study [19]. With an 80% power, α of 0.05 and 2-tailed test, 6 mice per group were recommended. Totally 36 mice were randomly divided into four groups: control, DM, experimental periodontitis (EP), and diabetes-associated periodontitis (DP) (n = 9/group). The 5–0 silk ligature was bound around the maxillary right first molar and remained in place for 14 days. The left first molar was not ligated as self-control for bone loss measurement. 7 days after the ligature was removed, the mice were euthanized and the maxillae were obtained for micro-computed tomography (micro-CT) and histological analysis. The maxillae were trimmed and fixed in 4% polyformaldehyde buffer for 48 h and scanned by micro-CT. After being decalcified with 20% EDTA at 4℃ for 30 days until the bone could be readily pierced, the specimens were routinely dehydrated and paraffin embedded for histochemical analysis. For micro-CT and histological measurement, the interproximal area between the maxillary first and second molars was selected as the region of interest (ROI).

### Cell preparation and stimulation

BMSCs were prepared according to previously published protocols [20]. The mandible was separated from male 6-week-old mice. Whole bone marrow cells were flushed out with α-MEM (C12571500BT, Gibco, NY, USA), supplemented with 1% penicillin/streptomycin and 10% FBS (BBP5, Moregate, New Zealand), and cultured in the incubator. The medium was changed every 2–3 days until the cells reach at 80% confluence for 7 days approximately. The cells were seeded into the plates at ratio of 1:3. The isolated cells were characterized by flow cytometric analysis of specific surface antigens, including CD29, CD44, CD105 and CD45. BMSCs were cultured in osteogenic or adipogenic induction medium for 21 days to detect the differentiation potential. The osteogenic induction medium was supplemented with 7% FBS, 1% penicillin/streptomycin, 10 mM glycerophosphate, 50 mg/l ascorbic acid and 100 nM dexamethasone. The adipogenic induction medium was supplemented with 10% FBS, 1 μM dexamethasone, 0.5 mM 3-isobutyl-1-methylxanthine, 0.1 M indomethacin and 10 μg/ml insulin.

The mouse pre-osteoblast line MC3T3 cells were purchased from Chinese Academy of Sciences (Shanghai, China). The cells were cultured in α-MEM, supplemented with 1% penicillin/streptomycin and 10% FBS. Cells were cultured with induction medium for osteogenic differentiation when they achieved 70–80% confluence.

BMSCs were exposed to AGEs (bs-1158P, Bioss, Beijing, China) at various amounts (0, 50, 100 and 150 μg/ml) for a period of 3 to 21 days. The induction medium was replaced every other day.

### Cell transfection with lentivirus (LV) and small interfering RNAs (siRNAs)

Three siRNAs targeting sclerostin (SOST, Gene ID: 74499) (siSOST) and negative control RNAs (siNC) were synthesized by Sangon (Shanghai, China). At 16–18 h of seeding, the BMSCs approximately at 80% confluence were transient transfected with siRNAs by Lipofectamine3000 (L3000015, Invitrogen, CA, USA) according to the manufacturer’s protocols.

The LV-Fto-RNAi (LV-shFTO) and LV-negative control (LV-shNC) were constructed by Genechem (Shanghai, China). BMSCs reached 30% confluent were infected with lentivirals by HitransG with MOL 10 according to the manufacturer’s instructions.

MC3T3 cells were chosen to establish stable FTO knockdown models, that were infected with LV-shFTO (Mol = 5) and were selected using puromycin (2 μg/ml). Real-Time quantitative Polymerase Chain Reaction (RT-qPCR) and Western blot (WB) were used to identify the efficiency of FTO knockdown. The primer sequences for PCR are provided in Additional file 1. The targeted sequences for siRNAs and shFTO are listed in Additional file 2.

Full details of the materials and methods are provided in Additional file 3.

## Results

### ***HFHG-induced T2DM damages alveolar bone regeneration in periodontitis by m***^***6***^***A modification***

**Fig. 1:**
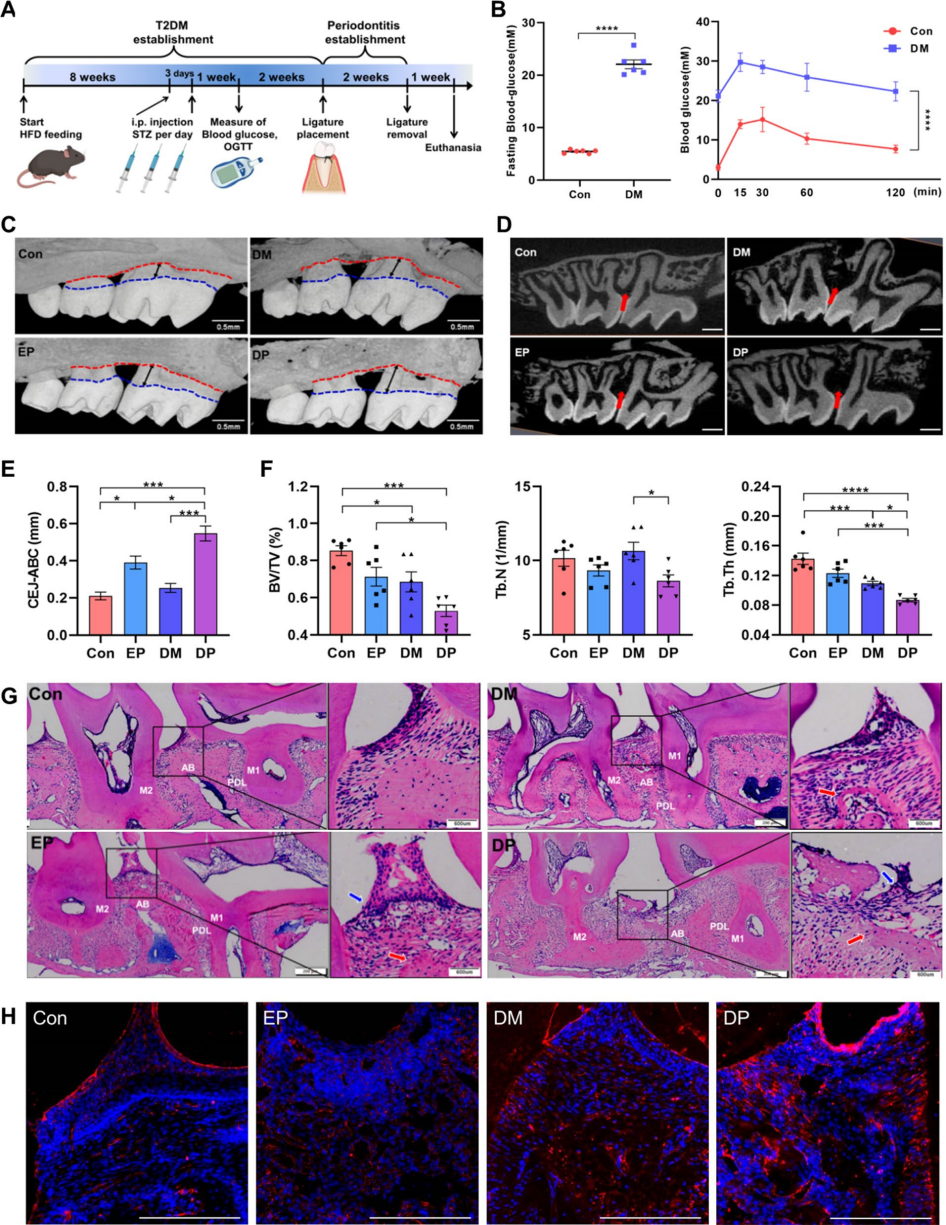
Diabetes mellitus (DM) aggravates periodontitis and alveolar bone loss by m^6^A methylation. **A** Scheme of the establishment of periodontitis with diabetes in mice. **B** Glucose levels of overnight-fasting and corresponding time points of the oral glucose tolerance test (OGTT) test one week after streptozotocin (STZ) administration (n = 6). **C**, **E** Representative micro–computed tomography (CT) 3-dimensional reconstruction shows the experimental periodontitis in normal and DM mice. Alveolar bone crests (ABC) are shown in red dotted lines, cemento-enamel junctions (CEJ) are shown in blue dotted lines. Scale bar = 500 μm. Quantification of alveolar bone loss, represented by distance of CEJ-ABC on buccal root surfaces of the maxillary first molar (Black arrow) (n = 6). *Con,* control; *DM,* diabetes mellitus; *EP,* experimental periodontitis; *DP,* diabetes-associated periodontitis. **D** Original micro-CT images of 2-dimensional slices on sagittal direction. The red arrow indicates the region of interest (ROI) with alveolar bone loss. Scale bar = 500 μm. **F** Quantitative analysis of bone volume/tissue volume (BV/TV), trabecular thickness (Tb·Th), and trabecular number (Tb.N) of alveolar bone in normal and DM mice with or without periodontitis (n = 6). **G** Representative images of hematoxylin and eosin (H&E) staining of periodontal tissue between maxillary first molar and second molar. Inflammatory infiltration (blue arrows), alveolar bone resorption (red arrows) are shown. Scale bar = 600 μm. *PDL*, periodontal ligament; *AB*, alveolar bone; *M1*, first molar; M2, second molar. **H** Representative immunofluorescence images of FTO at 200 × magnification (scale bar = 200 µm). Data are expressed as the mean ± SEM. ns, not significant. *P < 0.05. **P < 0.01. ***P < 0.001. ****P < 0.0001

The mice were fed with high fat/high glucose (HFHG) diet and their body weight reached 30–35 g at 8 weeks. They developed symptoms one week after receiving STZ intraperitoneally injection. The increased fasting blood glucose and abnormal glucose tolerance indicated that T2DM was established (Fig. 1A, B). Micro-computed tomography (CT) scan showed that the CEJ-ABC distance in both experimental periodontitis and diabetes-associated periodontitis groups were increased, and the alveolar bone loss in diabetes-associated periodontitis group was significant (Fig. 1C, E). Bone volume/tissue volume (BV/TV) and trabecular thickness (Tb.Th) of the DM group were lower than those in the control group, but the CEJ-ABC distance did not change significantly (Fig. 1D, F). The HE staining of the periodontal tissue showed that the gingival epithelium of control and diabetes groups were complete, no alveolar bone absorption was found, but the bone trabeculae in DM group were sparse. With regard to experimental periodontitis and diabetes-associated periodontitis groups, the integrity of the gingival epithelium was destroyed, the combined epithelium proliferated to the root, a large number of inflammatory cells infiltrated, and the alveolar bone was obviously absorbed. The destruction of periodontal tissue in diabetes-associated periodontitis group was more serious than that in experimental periodontitis group (Fig. 1G). The FTO expressions in alveolar bone were examined by immunofluorescence (IFC). Compared with that in control group, the diabetes-associated periodontitis group showed the largest increase in FTO expression, followed by that in the diabetes group (Fig. 1H).

### *N*^6^-methyladenosine-RNA immunoprecipitation sequence (MeRIP-Seq) reveals the m^6^A modification pattern, functional enrichment of differentially methylated genes and modification of target gene in alveolar bone of periodontitis with diabetes

**Fig. 2:**
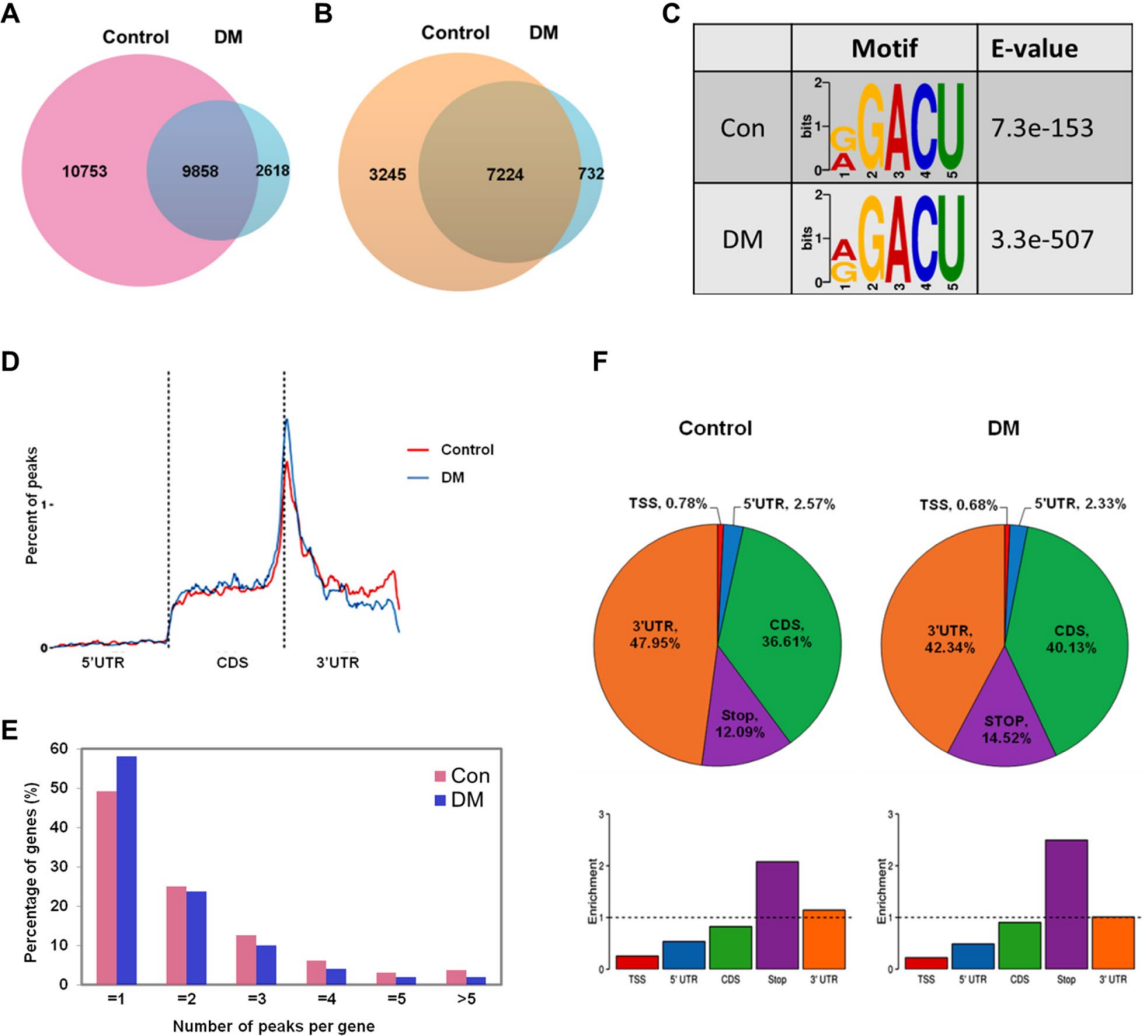
Overview of m^6^A methylation patterns in alveolar bone of normal and diabetes. **A**, **B** Venn diagram showing the overlap of m^6^A peaks number in two groups represents the m^6^A modification changes (**A**) and gene modification changes (**B**). The quantity of control-unique, DM-unique and common of m^6^A peaks and m^6^A-regulated genes are shown respectively. **C** The conserved consensus RGACU of m^6^A motif was identified in both control and DM groups. **D** Density curve depicts the distribution of m^6^A peaks along transcripts of two groups. **E** Percentage of genes with different numbers of m^6^A peaks in two groups. **F** Distribution of m^6^A peaks in the functional region of transcripts. The pie chart depicts the proportion distribution of m^6^A peaks on each region (top), and the histogram depicts the enrichment ratio of m^6^A peaks on the corresponding element (bottom)

Considering previous results suggesting that diabetes impaired the microstructure and reduced the repair and regeneration ability of alveolar bone, in order to dissect the m^6^A methylation mechanism of alveolar bone in diabetes, we conducted MeRIP-Seq to map the m^6^A modification and identify the critical m^6^A targets. The m^6^A-seq analysis identified 20611 m^6^A peaks representing 10469 gene transcripts in control and 12476 m^6^A peaks representing 7956 gene transcripts in DM, of which 9858 m^6^A peaks corresponding to 7224 gene transcripts were common between the two groups (Fig. 2A, B). The consensus sequence RGACU was significantly enriched in the m^6^A sites in both groups, which was consistent with previous research and indicated the sequence conservation of m^6^A motif (Fig. 2C). The accumulation of m^6^A peaks in different regions of mRNA was showed by density curve. The ordinate represented the proportion of peaks in the region at the corresponding position in relation to all peaks. The m^6^A peaks of two groups were particularly abundant near the end of coding sequences (CDS) and the beginning of 3ʹ untranslated region (3ʹUTR) (Fig. 2D). The m^6^A peaks distribution patterns on the functional region of mRNA were further analyzed based on the m^6^A-seq results. The pie chart showed the peaks distribution in 3'UTR region accounted for the largest proportion, and the histogram revealed the peaks were mostly enriched in the stop-codon. Meanwhile, similar patterns of m^6^A peaks distribution were found in control and DM groups (Fig. 2F). As Fig. 2E displayed, most genes have only one m^6^A modified peak. Especially in DM group, the proportion of genes containing unique peak modification increased than control.

**Fig. 3:**
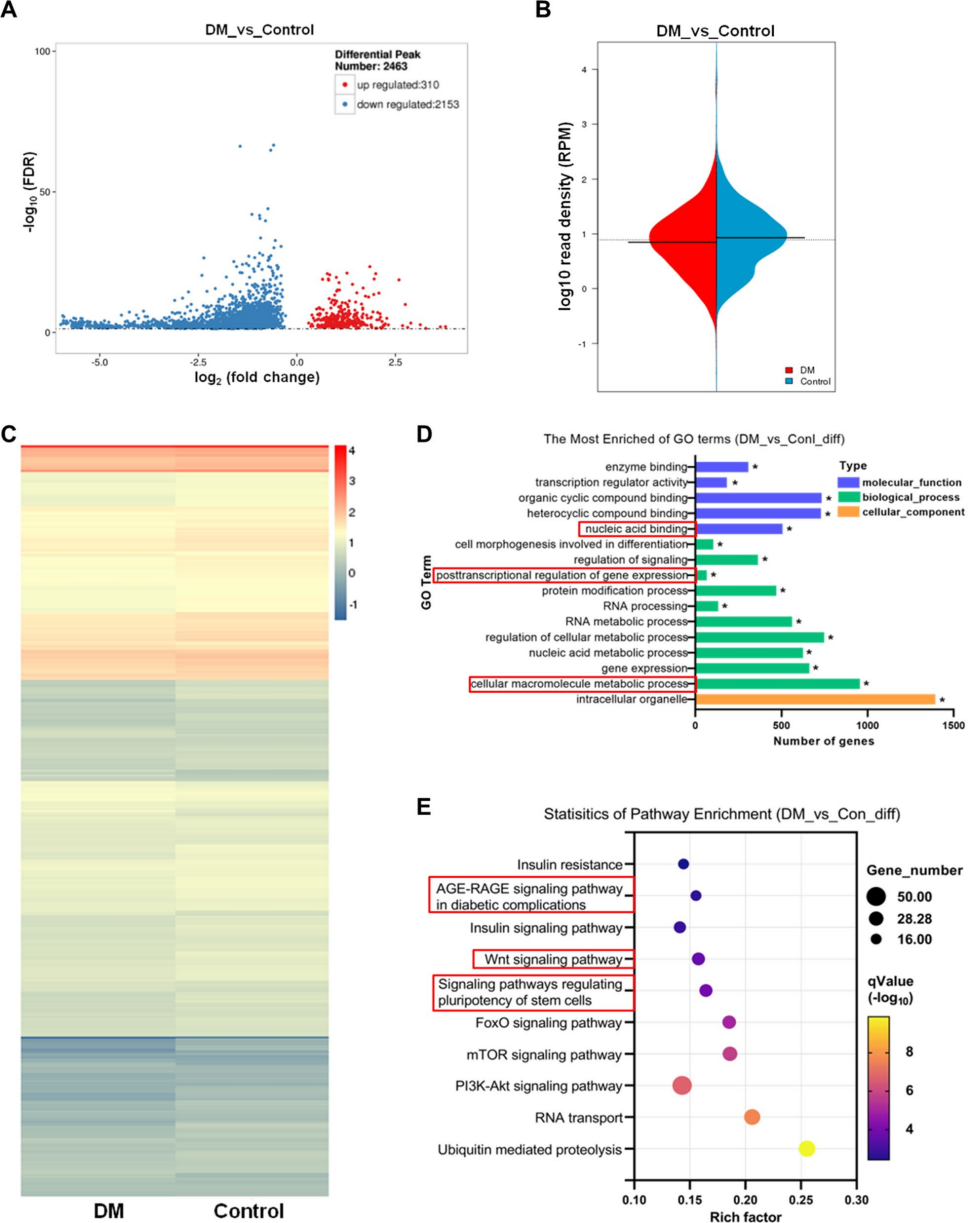
Biological functions and signaling pathways associated with differentially m^6^A methylated transcripts in alveolar bone with diabetes. **A** Volcano plot representation of the differential m^6^A peaks between the control and DM group. **B** Bean plot of differential m^6^A peak signal distribution. **C** Heat map of Differential peak signal distribution. **D** GO enrichment analysis of all genes related to differential m^6^A peaks. **E** KEGG pathway analysis of all differential m^6^A methylation modified genes

Volcano plot showed that DM had 310 differentially hypermethylated peaks and 2153 differentially hypomethylated peaks compared with control group (Fold change > 2 and p < 0.05) (Fig. 3A). Bean plot showed that the overall RPM distribution of the differential m^6^A peaks in control (blue part) and DM (red part) group was almost consistent, and the median and average values of RPM in DM group were lower than control group (Fig. 3B). The differential m^6^A peaks based on RPM counts were exhibited in the form of heat map (Fig. 3C). To determine the potential biological significance of changes in m^6^A modification associated with DM, we conducted GO and KEGG analysis of differentially methylated genes. The results of GO analysis revealed the differential m^6^A modified transcripts were particularly enriched in “cellular macromolecule metabolic process”, “posttranscriptional regulation of gene expression” and “nucleic acid binding” (Fig. 3D). Further, with respected to the KEGG analysis of genes with differential m^6^A peaks, we found that the genes were enriched in “Signaling pathways regulating pluripotency of stem cells” and “AGE-RAGE signaling pathway in diabetic complications”. The enrichment of PI3K-Akt, Wnt signaling pathway indicated the correlation with osteogenesis and bone homeostasis (Fig. 3E).

### ***AGEs lead to compromised osteogenesis of BMSCs and reduce m***^***6***^***A methylation level ***in vitro

**Fig. 4:**
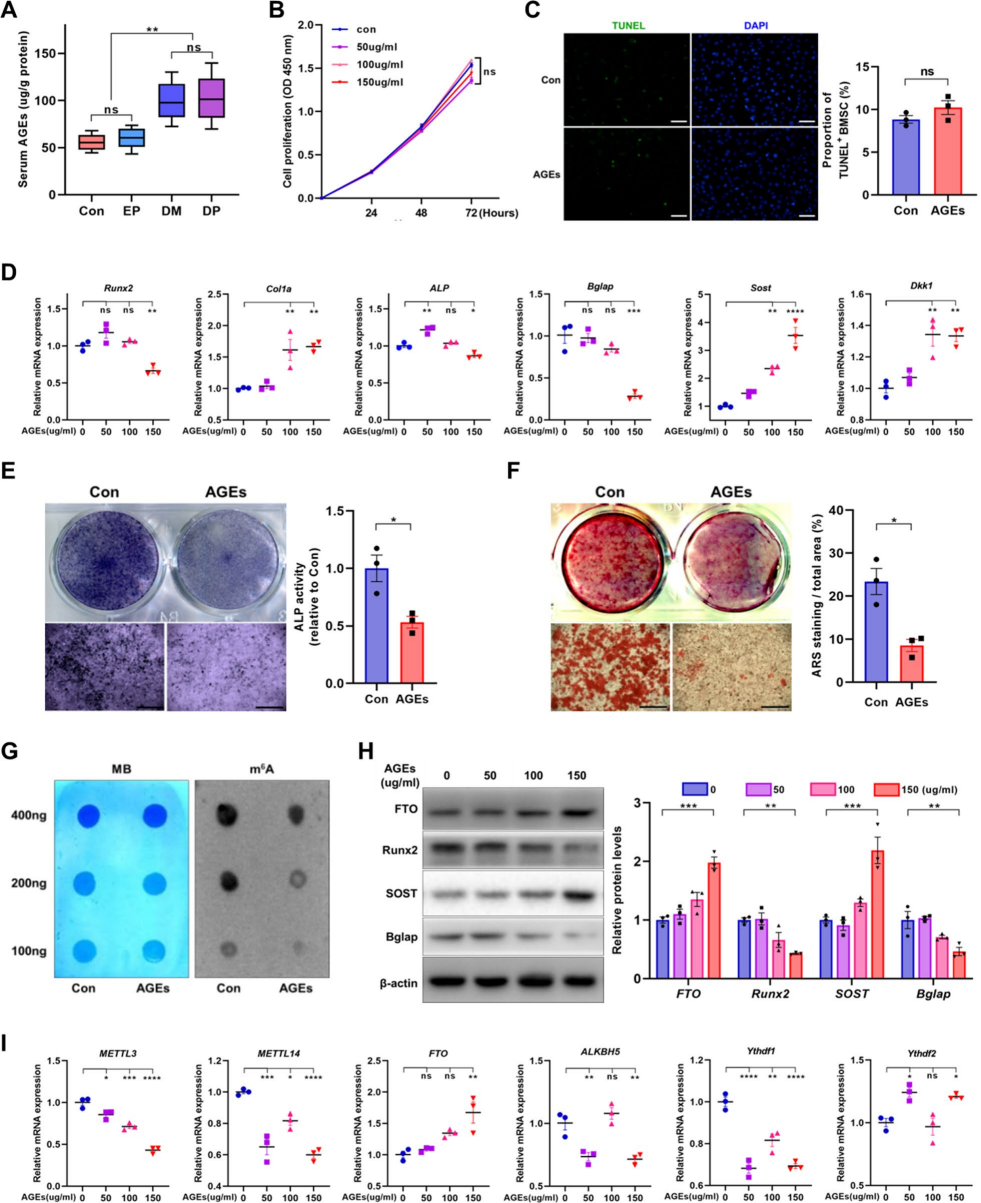
AGEs affect the osteogenic differentiation and m^6^A modification level of BMSCs. **A** ELISA quantification of serum AGEs level of control and DM mice with or without experimental periodontitis (n = 6). **B** Proliferation of BMSCs stimulated with different doses of AGEs (0, 50, 100 or 150 µg/ml) was determined by CCK-8 assay (n = 3). **C** Detection of apoptotic BMSCs through DAPI and TUNEL staining. The percentage of TUNEL-positive BMSCs was counted from three random microscopic fields (n = 3). Scale bar = 200 μm. **D** Real-Time quantitative Polymerase Chain Reaction (RT-qPCR) analysis of the expression of Runx2, Col1a, ALP, Bglap, SOST and Dkk1 in BMSCs untreated or treated with AGEs under osteogenic induction for 3 days (n = 3). **E** Representative images of alkaline phosphatase (ALP) staining and quantification of ALP activity in BMSCs at day 7 after exposure to osteogenic induction with or without AGEs (n = 3). Scale bars = 1000 μm. **F** Representative images of Alizarin red S (ARS) staining and quantification of mineralization nodules in BMSCs at day 21 after exposure to osteogenic induction with or without AGEs (n = 3). Scale bars = 1000 μm. **G** The m^6^A methylation levels in BMSCs at day 10 after exposure to osteogenic induction with or without AGEs were detected by m^6^A dot blot assays. Input RNA was assessed by methylene blue staining (left panel), and the level of m^6^A modification was determined by the intensity of dot immunoblotting (right panel). **H** Western blot (WB) analysis of the protein level of Runx2, Bglap, SOST and FTO in BMSCs untreated or treated with AGEs under osteogenic induction for 5 days (n = 3). **I** RT-qPCR analysis of the expression of Mettl3, Mettl14, FTO, Alkbh5, Ythdf1 and Ythdf2 in BMSCs untreated or treated with AGEs under osteogenic induction for 3 days (n = 3). Data are expressed as the mean ± SEM. ns, not significant. *P < 0.05. **P < 0.01. ***P < 0.001. ****P < 0.0001

Characterization of cultured BMSCs was identified by flow cytometry and differentiation induction (Additional file 4). The serum AGEs levels in mice were quantified and significantly increased in the DM mice compared to control (Fig. 4A). To explore the influence of diabetes microenvironment on periodontal tissue, different concentrations of AGEs were used to stimulate BMSCs. Cell counting kit-8 (CCK8) suggested that various concentrations of AGEs had no significant influence on cell growth within 72 h (Fig. 4B). TdT-mediated dUTP nick-end labeling (TUNEL) staining showed that AGEs (150 μg/ml) did not significantly affect the apoptosis of BMSCs (Fig. 4C). The results of Real-Time quantitative Polymerase Chain Reaction (RT-qPCR) showed that the mRNA expression of RUNX family transcription factor 2 (Runx2) and bone gamma-carboxyglutamate protein (Bglap) were inhibited at different degrees, while the expression of sclerostin (SOST) and dickkopf WNT signaling pathway inhibitor 1 (Dkk1) were up-regulated, suggesting that AGEs might affect the osteogenic differentiation of BMSCs, and 150 μg/ml was selected as the working concentration for subsequent experiments (Fig. 4D). Alkaline phosphatase (ALP) staining and ALP activity detection suggested that AGEs inhibited the synthesis and activity of ALP (Fig. 4E). Alizarin red S (ARS) staining showed that AGEs inhibited the calcium deposition of BMSCs (Fig. 4F). Dot blot assay presented the m^6^A methylation level of total RNA in BMSCs stimulated by AGEs decreased (Fig. 4G). The expression of the m^6^A-methylation related enzymes were verified by qPCR, METTL3, METTL14 and ALKBH5 were down-regulated, while the expression of FTO was up-regulated (Fig. 4I). WB verified the decrease of Runx2, Bglap and the increase of SOST and FTO in AGEs treatment group (Fig. 4H), which suggested that FTO might have a regulatory function in the process of AGEs prevented BMSCs from differentiating into osteoblasts. The uncropped blots of WB are provided in Additional file 5.

### FTO knockdown reverses the impaired osteogenesis of BMSCs caused by AGEs

**Fig. 5:**
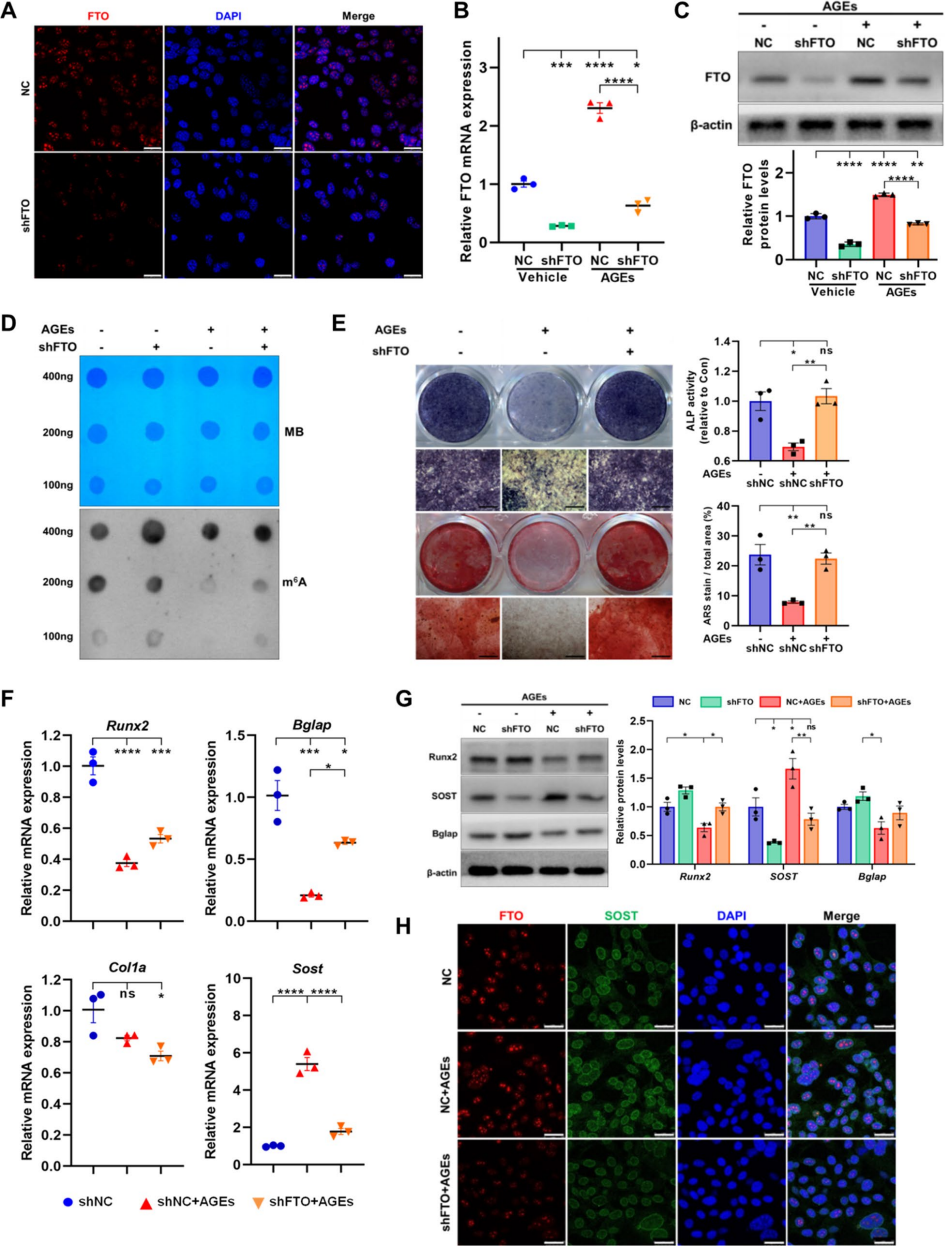
The m^6^A demethylase FTO negatively regulates the osteogenesis of BMSCs stimulated with AGEs. **A** Immunofluorescence assay showed the protein level and location of FTO in negative control (shNC) and FTO-knockdown (shFTO) BMSCs. Scale bars = 25 μm. **B**, **C** RT-qPCR and WB analysis of FTO expression in negative control (shNC) and FTO-knockdown (shFTO) BMSCs treated with or without AGEs (n = 3). **D** M^6^A dot blot analysis revealed the m^6^A methylation levels in BMSCs infected with shFTO under the exposure to AGEs or not. Methylene blue stain was utilized as loading control. **E** Representative images of ALP and ARS staining and quantification of ALP activity and mineralization nodules in BMSCs infected with shFTO under the exposure to AGEs or not after osteogenic induction for 7–21 days (n = 3). Scale bars = 400 μm. **F** RT-qPCR analysis of the expression of Runx2, Bglap, Col1a and SOST in BMSCs infected with shNC or shFTO under the exposure to AGEs or not after 3 days of osteogenic induction (n = 3). **G** Western blot analysis of the expression of Runx2, Bglap and SOST in BMSCs infected with shNC or shFTO under the exposure to AGEs or not after osteogenic induction for 5 days (n = 3). **H** Immunofluorescence assay showed the protein level and location of FTO and SOST in BMSCs infected with shNC or shFTO under the exposure to AGEs or not. Scale bars = 25 μm. Data are expressed as the mean ± SEM. ns, not significant. *P < 0.05. **P < 0.01. ***P < 0.001. ****P < 0.0001

To observe the role of FTO-mediated m^6^A modification in AGEs impairing the osteogenic differentiation of BMSCs, lentivirus-FTO-shRNA-PURO (LV-shFTO) were constructed to knockdown the expression of FTO. Immunofluorescence, qPCR and WB confirmed the knockdown efficiency of LV-shFTO, by which effectively reduced the upregulation of FTO caused by AGEs stimulation (Fig. 5A–C). As expected, dot blot showed that FTO knockdown increased the total m^6^A level in BMSCs, confirming the biological function of demethylase FTO (Fig. 5D). ALP activity and ALP, ARS staining showed that FTO knockdown reduced the inhibition of bone formation potential of BMSCs stimulated by AGEs (Fig. 5E). The qPCR and WB also confirmed that FTO knockdown reversed the down-regulation of Runx2 and Bglap expression caused by AGEs, and inhibited the upregulation of SOST induced by AGEs (Fig. 5F, G). Double-label immunofluorescence proved that AGEs increased the expression of intracellular FTO and SOST, but FTO knockdown could reduce the level of SOST, suggesting that FTO knockdown might partially alleviated the inhibition of osteogenesis of BMSCs induced by AGEs (Fig. 5H).

### *Sclerostin interference relieves the inhibition of osteogenesis induced by AGEs *via* Wnt signaling pathway*

**Fig. 6:**
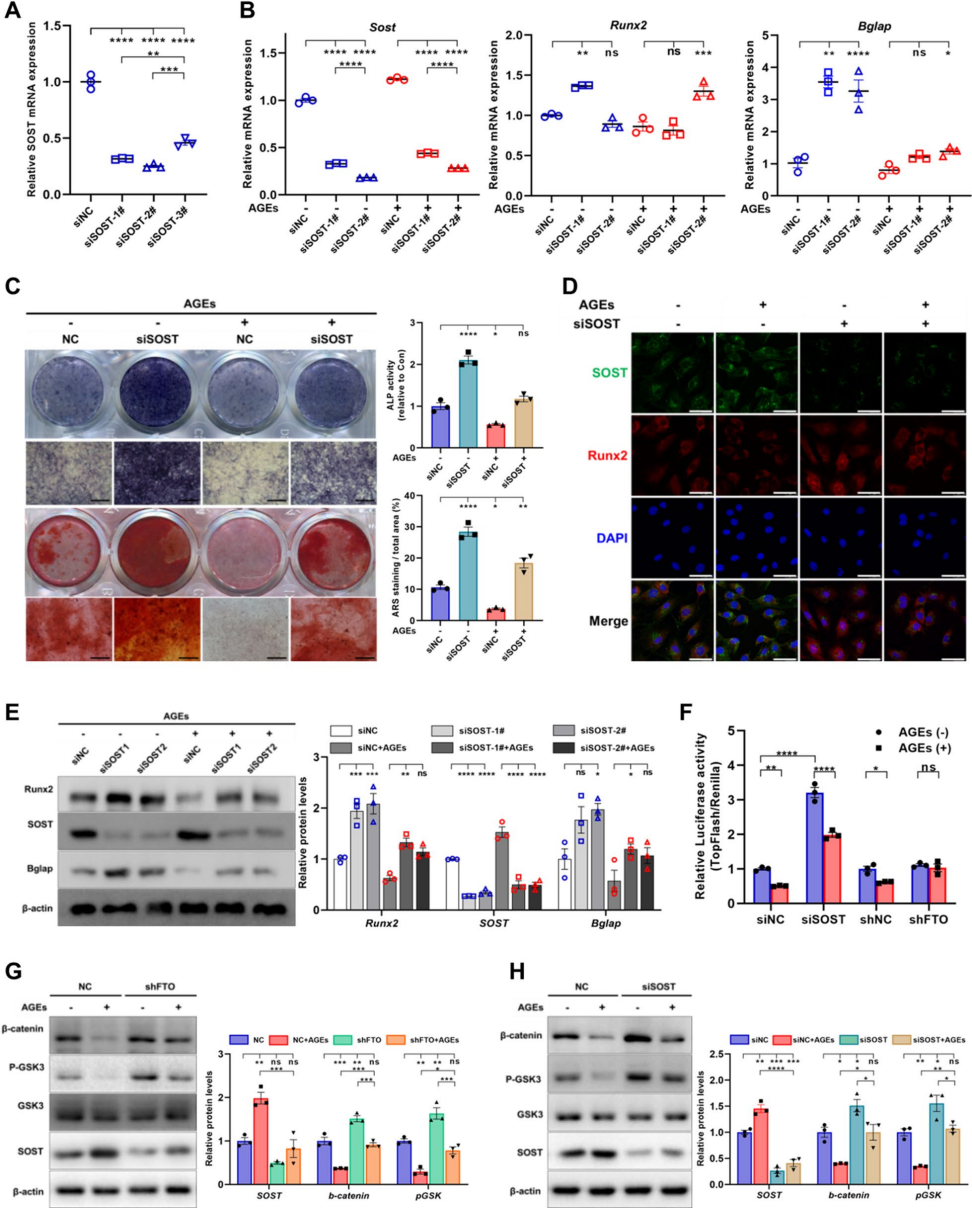
Sclerostin (SOST) interference ameliorates the impaired osteogenesis of BMSCs induced by AGEs via Wnt signaling. **A** RT-qPCR analysis of the mRNA expression of SOST in BMSCs transfected with small interfering RNAs targeting sclerostin (siSOST) or negative control (siNC) (n = 3). **B** RT-qPCR analysis of the expression of Runx2, Bglap and SOST in BMSCs transfected with siSOST or siNC under exposure to AGEs or not after osteogenic induction for 3 days (n = 3). **C** Representative images of ALP and ARS staining and quantification of ALP activity and mineralization nodules in BMSCs transfected with siSOST or siNC under exposure to AGEs or not after 7–21 days of osteogenic induction (n = 3). Scale bars = 400 μm. **D** Immunofluorescence assay showed the protein level and location of SOST and Runx2 in BMSCs transfected with siSOST or siNC under exposure to AGEs or not. Scale bars = 50 μm. **E** Western blot analysis of the expression of Runx2, Bglap and SOST in BMSCs transfected with siSOST or siNC under exposure to AGEs or not after osteogenic induction for 5 days (n = 3). **F** TOPflash/Renilla activity in MC3T3 cells treated with siNC, siSOST, shNC and shFTO under exposure to AGEs or not (n = 3). **G** Protein levels of phos-GSK3β, total GSK3β, β-catenin and SOST in BMSCs infected with shNC or shFTO under exposure to AGEs or not (n = 3). **H** Protein levels of Wnt/β-catenin pathway in BMSCs transfected with siNC or siSOST under exposure to AGEs or not (n = 3). Data are expressed as the mean ± SEM. ns, not significant. *P < 0.05. **P < 0.01. ***P < 0.001. ****P < 0.0001

To clarify the role of SOST in AGEs inhibiting osteogenic differentiation of BMSCs, siRNAs were used to silence the SOST expression. The siRNAs targeting SOST (siSOST) were verified according to qPCR and used for subsequent experiments (Fig. 6A). RT-qPCR and WB showed that SOST interference increased the expression of Runx2 and Bglap in BMSCs of control. In BMSCs treated with AGEs, siSOST rescued the expression of Runx2, a marker gene of early osteogenesis, but had limited effect on the recovery of Bglap, a marker gene of mineralization (Fig. 6B, E). ALP activity assay, ALP and ARS staining exhibited that SOST interference improved the alkaline phosphatase activity and mineralized deposition of BMSCs. In BMSCs treated with AGEs, SOST siRNAs partially restored the compromised osteogenic potential (Fig. 6C). Double-label immunofluorescence displayed that AGEs increased the intracellular SOST and decreased Runx2, siSOST reduced the SOST expression and alleviated the suppression of Runx2 caused by AGEs (Fig. 6D). Since SOST is an inhibitor of Wnt signaling and its expression was induced by AGEs, we next used TOPFlash luciferase assay to detect the SOST interference or FTO knockdown on the impact of β-catenin activation. Data suggested that AGEs suppressed TOPFlash/Renilla activity in negative control groups and this activity was increased in presence of siSOST, and shFTO attenuated the suppressive effect of AGEs exposure (Fig. 6F). WB was performed to verify the effect of FTO knockdown and SOST interference on Wnt pathway respectively. It was found that AGEs inhibited GSK phosphorylation and reduced β-catenin level, while FTO-knockdown and SOST interference partially relieved inhibition of Wnt pathway induced by AGEs (Fig. 6G, H).

### ***FTO regulates stability of sclerostin transcripts in an m***^***6***^***A-dependent manner***

**Fig. 7:**
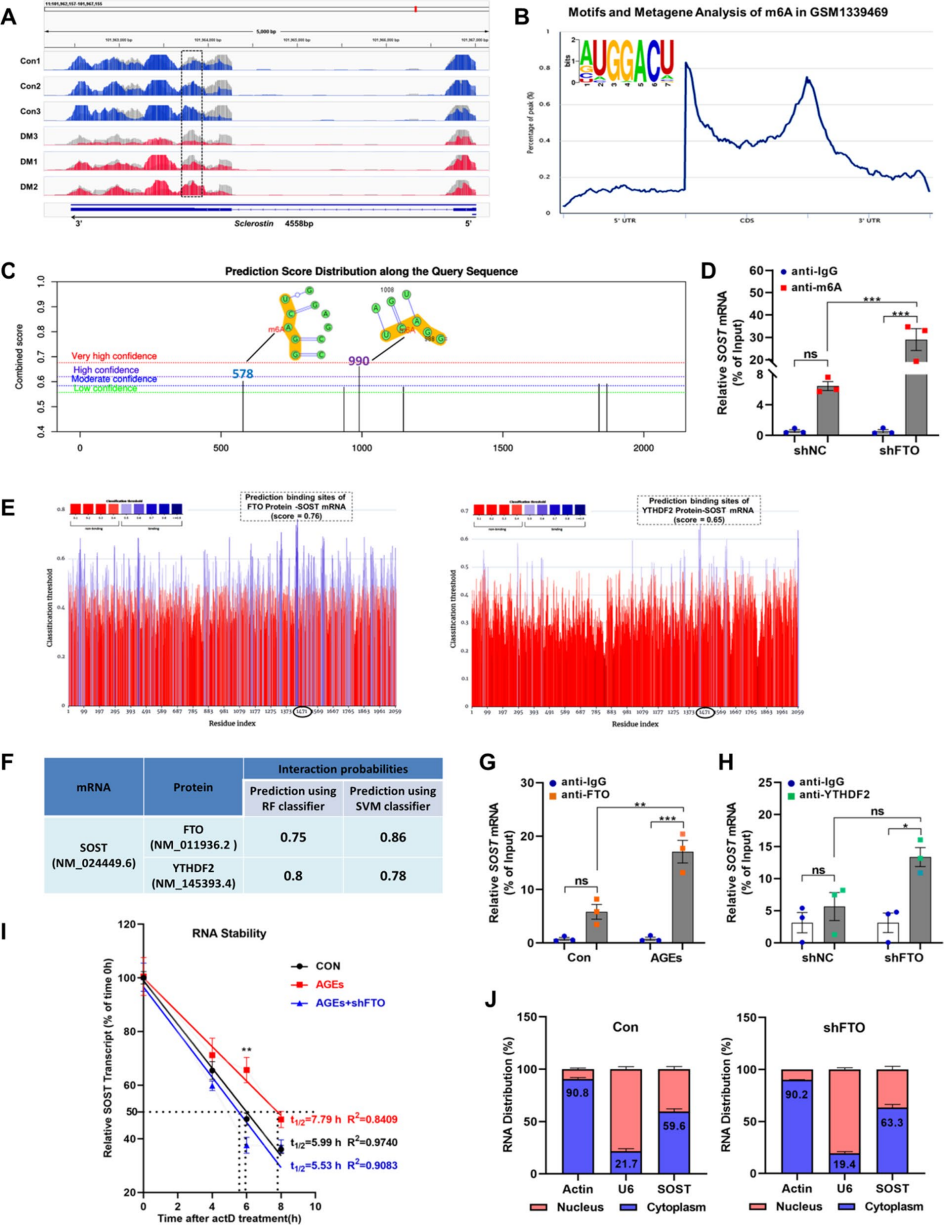
FTO-mediated m^6^A modification in BMSCs regulates sclerostin stability. **A** M^6^A-Seq identified the m^6^A site in CDS-3’-UTR junction region of SOST transcript. **B** M^6^A consensus motif and metagene analysis of the SOST transcript. **C** SRAMP prediction results of m^6^A sites on SOST transcript. **D** MC3T3 cells were stably infected with FTO-shRNA or negative control-shRNA by lentivirus vector, the m^6^A modification of SOST transcripts were detected by m^6^A immunoprecipitation (MeRIP)-qPCR (n = 3). **E** PRIdictor Database displays the potential Protein-RNA binding sites of FTO and YTHDF2 on SOST transcript. **F** RPISeq Prediction estimates the Interaction probability of FTO and YTHDF2 protein to SOST transcript. **G** RNA immunoprecipitation-qPCR assay evidenced the SOST transcript enrichment precipitated by anti-FTO antibody in BMSCs treated with or without AGEs. IgG acted as the blank control (n = 3). **H** RNA immunoprecipitation-qPCR assay evidenced the SOST transcript enrichment precipitated by anti-YTHDF2 antibody in MC3T3 cells stably infected with LV-shNC or LV-shFTO. IgG acted as blank control (n = 3). **I** RNA stability assay showed the half-life (t_1/2_) of SOST mRNA in BMSCs treated with AGEs exposure and FTO knockdown (n = 3). **J** The relative level of SOST transcripts in subcellular fractions of BMSCs infected with LV-shNC or LV-shFTO was detected by RT-qPCR. β-actin and U6 were employed as cytoplasmic and nuclear loading controls, respectively (n = 3). Data are expressed as the mean ± SEM. ns, not significant. *P < 0.05. **P < 0.01. ***P < 0.001

To elucidate the underlying mechanism by which FTO regulates the osteogenic differentiation of BMSCs, the enrichment of m^6^A peaks in SOST transcripts from MeRIP-seq data was visualized by Integrative Genomics Viewer (IGV). Notably, the m^6^A modification in SOST was markedly decreased in alveolar bone of diabetes (Fig. 7A). Bioinformatics analysis was performed to investigate the interaction between m^6^A modification and demethylase FTO to the SOST transcript. The m^6^A motif and metagene analysis was detected using RMBase V2.0 (http://rna.sysu.edu.cn/rmbase/). It revealed that the sites of m^6^A modification on SOST mRNA were GGACU and mainly situated in the CDS-3ʹUTR junction region, which was consistent with our IGV results (Fig. 7B). Then, potential m^6^A binding sites on SOST mRNA were further investigated using the online m^6^A site prediction tool SRAMP (http://www.cuilab.cn/sramp/). SRAMP analysis revealed that two potential m^6^A sites with high prediction scores were located in the CDS-3ʹUTR junction of SOST. Next, we selected the sequence of the CDS-3ʹUTR junction as a template to design primers for m^6^A-RIP-qPCR (Fig. 7C). Methylated RNA immunoprecipitation qPCR (MeRIP-qPCR) was performed in MC3T3 cells stable infected with LV-shFTO and indicated that m^6^A-specific antibodies significantly enriched on SOST transcripts compared with the IgG, which verified the predicted m^6^A sites existed on the selected region. FTO knockdown significantly increased the m^6^A enrichment in SOST transcripts compared with the cells infected with LV-shNC, suggesting the m^6^A modification of SOST transcripts was regulated by FTO (Fig. 7D). To determine whether SOST is regulated by FTO and YTHDF2, RPISeq (http://pridb.gdcb.iastate.edu/RPISeq) and PRIdictor (http://www.rnainter.org/PRIdictor/) were used to predict the interaction probability and binding sites. RPISeq analysis showed that the binding probability of FTO and YTHDF2 protein to SOST transcript were more than 0.5, which was considered to be highly possible for direct regulation (Fig. 7F). PRIdictor analysis showed FTO had higher binding residues than YTHDF2 (559 vs. 77), and the protein-binding sites location in 3ʹUTR was surprisingly consistent (Fig. 7E). We concentrated on the predicted binding sites and created primers to ensure the anticipated binding sites were included in the target sequence. Subsequently, the RIP-qPCR identified SOST transcripts as FTO substrates, particularly under AGEs exposure condition (Fig. 7G). Furthermore, YTHDF2-RIP-qPCR was performed and found that the SOST transcripts were not significantly enriched with YTHDF2-antibody or IgG in shNC group. However, FTO-knockdown increased SOST transcripts enrich to YTHDF2 compared to IgG, but not significantly affected the SOST transcripts enrichment to YTHDF2 compared to shNC group (Fig. 7H). In addition, RNA stability assay demonstrated that the half-lives of the SOST mRNA in control, AGEs and AGEs with shFTO group were 5.99, 7.79 and 5.53 h respectively, suggesting that AGEs exposure retard the decay of SOST mRNA, while FTO knockdown reduced the stability of the mRNA (Fig. 7I). Moreover, the subcellular distribution assay revealed that FTO knockdown had no discernible impact on the distribution of SOST transcripts in the cytoplasm and nucleus, indicating that FTO knockdown had no significant effect on the nuclear export of SOST transcripts (Fig. 7J).

## Discussion

The bone homeostasis disorder caused by diabetes-associated periodontitis manifested as increased bone loss and insufficient bone regeneration [21]. Micro-CT validated that diabetes did not increase the CEJ-ABC distance, but affected the microstructure of alveolar bone, and in periodontitis, diabetes aggravated alveolar bone loss, which is consistent with previous reports [22]. Hyperglycemia led irreversible AGEs formation through non-enzymatic glycosylation and oxidation of proteins and lipids. The AGEs level in gingival crevicular fluid of patients with periodontitis and diabetes was significantly increased [23]. AGEs induced inflammation by activating inflammasomes, initiated apoptosis and autophagy by aggravating oxidative stress and mitochondria-mediated pathway in periodontal ligament cells (PDLCs) [24–26]. Blocking AGEs/RAGE pathway could attenuate bone loss and inflammatory factor production in periodontitis with diabetes [27]. Accumulation of AGEs is an independent risk factor for BMD reduction and fracture in elderly patients with diabetes [28]. AGEs significantly decreased the mRNA expression of osteogenic related genes in BMSCs [29]. Our findings verified that AGEs inhibited the osteogenesis and m^6^A methylation level of BMSCs, indicating that AGEs may impair osteogenesis by RNA post-transcriptional regulation.

Recently, growing evidence has indicated that m^6^A methylation modification is closely related to diabetic complications. The expression of FTO in tissue and cells of T2DM patients and animal models were upregulated, which is involved in diabetic complications [12, 30–32]. HFD increased FTO expression and disrupted glycolipid metabolism [13, 33]. FTO was increased in retinal pigment epithelial and HepG2 cells induced by high glucose, affecting glucose metabolism and resulting in pyroptosis [12, 34]. Studies reported that m^6^A modification mediated the differentiation of BMSCs by regulating the transcription, translation and degradation of osteogenic related genes and also regulated the proliferation, differentiation and apoptosis of osteoblasts, chondrocytes, osteoclasts and dental pulp cells [16, 35]. At present, the role of FTO in osteogenesis is still controversial. Studies reported that FTO was up-regulated in osteoporosis patient and OVX mice. FTO was deceased in BMSCs during osteogenesis and FTO knockdown could enhance the osteogenic differentiation [18, 36–39]. Conversely, studies observed that FTO was up-regulated during differentiation of MSCs into osteoblasts [40]. FTO protected cells from genotoxic damage by enhancing the mRNA stability and maintained the differentiation potential [41]. Results in this study exhibited that FTO knockdown had no significant effect on the differentiation of BMSCs cultured in osteogenic induction medium, but for BMSCs exposed to AGEs, FTO-knockdown ameliorated the osteogenesis suppression caused by AGEs.

The GO and KEGG analysis revealed the differentially methylated transcripts were closely associated with the AGEs exposure, differentiation of BMSCs and posttranscriptional regulation of gene expression. To further elucidate the epigenetic mechanism, we speculated that SOST might be a target of FTO-mediated m^6^A methylation through the data of MeRIP-seq analysis and bioinformatics prediction. RIP-qPCR suggested that AGEs-induced FTO reduced m^6^A methylation of SOST transcripts. YTHDF2 participated in the recognition of m^6^A-modificatiion on SOST transcript as a reader and modulated the stability of SOST mRNA. Cumulative SOST might inhibit the osteogenesis via the negative regulation of Wnt signaling.

Sclerostin is a secreted glycoprotein that blocks the canonical Wnt/β-Catenin pathway and has gradually emerged as a novel target for treatment of skeletal disease [42]. Clinical trials have confirmed that the increased circulating SOST and accumulation of AGEs were potential mechanisms of low bone turnover and raised risk of fracture in T2DM [43]. HFD reduced bone formation and increased SOST expression in mice [44]. AGEs could increase SOST expression and induce dysfunction and apoptosis of osteocyte [45]. This study found that SOST may influence the expression of Runx2 in BMSCs treated with AGEs via the Wnt/β-catenin pathway. SOST interference increased the osteogenesis of BMSCs and also alleviated the damage caused by AGEs, indicating the application of Sost-inhibitor in the future may help gain more insights.

**Fig. 8:**
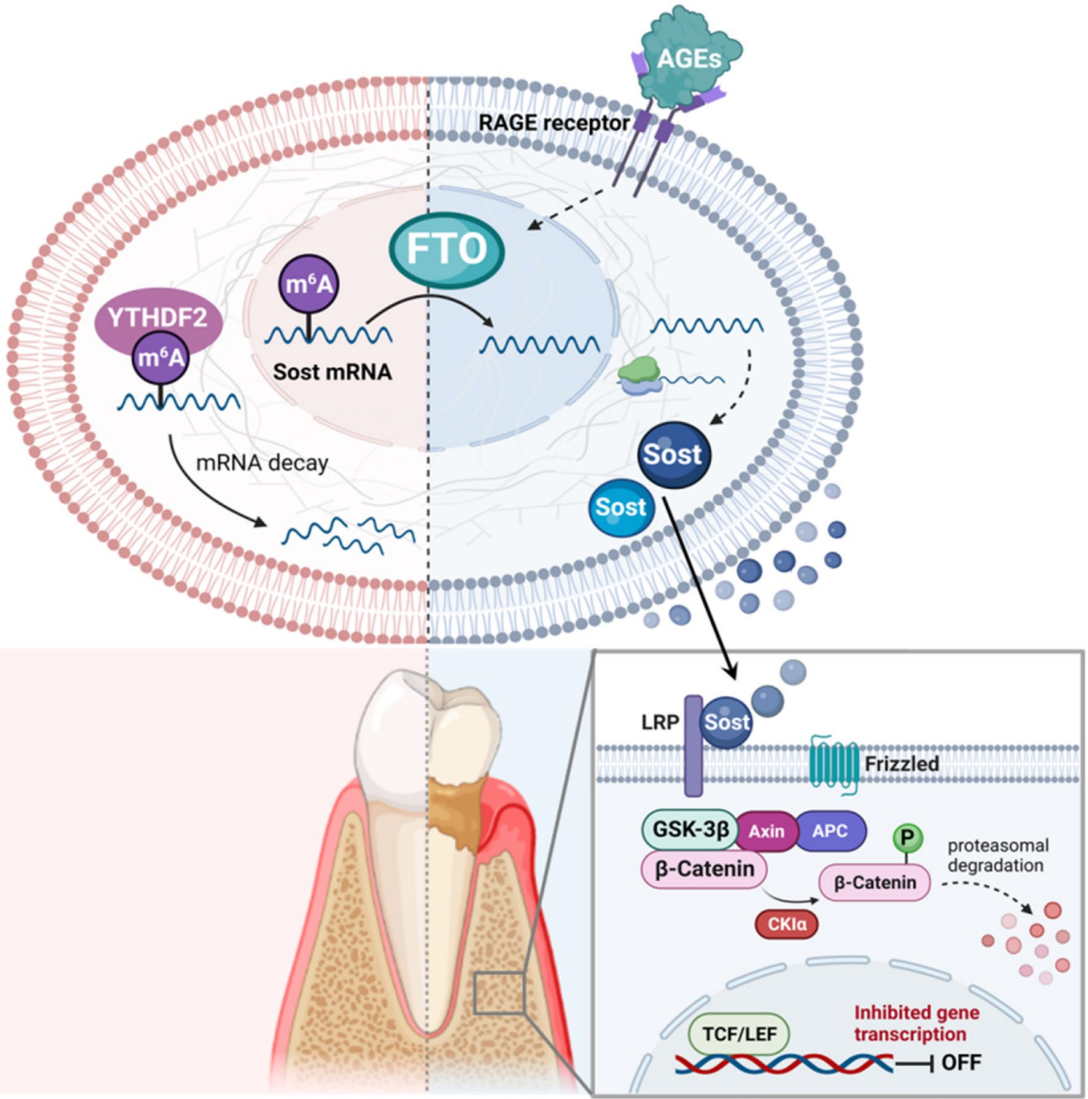
Schematic diagram (created with biorender.com) illustrates FTO-mediated m^6^A modification of SOST transcripts contribute to the impaired osteogenesis of BMSCs caused by AGEs via Wnt signaling

In conclusion, we found FTO was up-regulated by the AGEs stimulation and FTO-knockdown protected the osteogenic differentiation of BMSCs exposed to AGEs. FTO regulated the m^6^A modification of SOST transcripts, increased mRNA stability with recognition by YTHDF2, inhibited Wnt signaling pathway and impaired cell osteogenesis (Fig. 8). Considering the role of AGEs and m^6^A methylation in diabetic complications and bone homeostasis, this study provides potential strategy for bone regeneration treatment of periodontitis with diabetes.

### Supplementary Information

**Additional file 1:** Primer sequences to measure the mRNA levels by qPCR.

**Additional file 2:** The targeted sequences for siRNAs and shFTO.

**Additional file 3:** Supplementary materials and methods.

**Additional file 4:** The culture and characterization of BMSCs.

**Additional file 5:** The uncropped blots of WB.

## Data Availability

The datasets used and/or analyzed during the current study are available from the corresponding author on reasonable request.
