# Supplementary material for: Advanced glycation end products impair bone marrow mesenchymal stem cells osteogenesis in periodontitis with diabetes via FTO-mediated N6-methyladenosine modification of sclerostin
Source: J Transl Med. 2023 Nov 4;21:781. doi: 10.1186/s12967-023-04630-5 (PMC10625275; doi:10.1186/s12967-023-04630-5)
Supplement: Supplementary file 1 — Additional file 1: Primer sequences to measure the mRNA levels by qPCR. [file 12967_2023_4630_MOESM1_ESM.docx]

**Additional file 1: Primer sequences to measure the mRNA levels by qPCR**

**Table S1. Primer sequences to measure the mRNA levels by qPCR**

| **Gene** | **Forward Primer sequences(5′-3′)** | **Reverse Primer sequences(5′-3′)** |
| --- | --- | --- |
| Runx2 | GGGAACCAAGAAGGCACAGA | GGATGAGGAATGCGCCCTAA |
| Col1a | CCCTGGTCCCTCTGGAAATG | GGACCTTTGCCCCCTTCTTT |
| Alpl | ATCGACGTGATCATGGGTGG | TGGGAATGCTTGTGTCTGGG |
| Bglap | ACCTCACAGATGCCAAGCC | GCCGGAGTCTGTTCACTACC |
| SOST | GGTGGAAGGGCCAGAAATCA | AGAAATGTGTCCGTGGGTGG |
| Dkk1 | TCTCTATGAGGGCGGGAACA | TTTCGGCAAGCCAGACAGAT |
| Mettl3 | CTGGGCACTTGGATTTAAGGAA | TGAGAGGTGGTGTAGCAACTT |
| Mettl14 | TCTGGGGAAGGATTGGACCT | CCTTTGATCCCCATCAGGCA |
| FTO | GAGCAGCCTACAACGTGACT | GAAGCTGGACTCGTCCTCAC |
| Alkbh5 | GCGGTCATCAACGACTACCA | AACTTGCAGCCGAAGCAAAG |
| Ythdf1 | CTGCAGTTAAGACGGTGGGT | TAGCAATGGCTGCCCATGAA |
| Ythdf2 | CAGGCAAGGCCGAATAATGC | TCTCCGTTGCTCAGTTGTCC |
| Actb | CAGCCTTCCTTCTTGGGTAT | TGGCATAGAGGTCTTTACGG |
