# Supplementary material for: Advanced glycation end products impair bone marrow mesenchymal stem cells osteogenesis in periodontitis with diabetes via FTO-mediated N6-methyladenosine modification of sclerostin
Source: J Transl Med. 2023 Nov 4;21:781. doi: 10.1186/s12967-023-04630-5 (PMC10625275; doi:10.1186/s12967-023-04630-5)
Supplement: Supplementary file 2 — Additional file 2: The targeted sequences for siRNAs and shFTO. [file 12967_2023_4630_MOESM2_ESM.docx]

**Additional file 2: The targeted sequences for siRNAs and shFTO**

**Table S2.**  **The targeted sequences for siRNAs and shFTO**

| Target Gene | sequence |
| --- | --- |
| ***siSOST-1#*** | 5’- GAGAACAACCAGACCAUGAACTT-3’; |
| ***siSOST-2#*** | 5’-AGCUGGAGAACGCCUACUAGATT-3’ |
| ***siSOST-3#*** | 5’-GUGUUAAUAUCGCUUUGUGAAGTT-3’. |
| ***LV-Fto-RNAi*** | GTCTCGTTGAAATCCTTTGAT |
|  |  |
