# Supplementary material for: Advanced glycation end products impair bone marrow mesenchymal stem cells osteogenesis in periodontitis with diabetes via FTO-mediated N6-methyladenosine modification of sclerostin
Source: J Transl Med. 2023 Nov 4;21:781. doi: 10.1186/s12967-023-04630-5 (PMC10625275; doi:10.1186/s12967-023-04630-5)
Supplement: Supplementary file 3 — Additional file 3: Supplementary materials and methods. [file 12967_2023_4630_MOESM3_ESM.docx]

**Additional file 3: Supplementary materials and methods**

**Supplementary Materials and Methods**

**Fasting blood glucose and oral glucose tolerance test (OGTT)**

The mice were orally administered with 10% glucose solution (1.5g/kg body weight of glucose) after over-night fasting. Tail vein blood samples were obtained before and after treatment for 15, 30, 60 and 120 minutes to measure the blood glucose level. After the experiment, feed and water were supplemented immediately.

**Micro-computed tomography (micro-CT) analysis**

At the end of each experimental period, mice were euthanasia by 100% CO_2_ inhalation. The maxillae were dissected in half, fixed in 4% paraformaldehyde for 48h and scanned by micro-CT Scanner (vivaCT 40, SCANCO Medical AG, Switzerland). Scans were set to 70 kV, 114µA and spatial resolution of 10µm. Three dimensional models were constructed with Amira software (Thermo Fisher Scientific, USA). The distance between the cement-enamel junction (CEJ) and the alveolar bone crest (ABC) was measured to asses bone resorption. The interproximal area between the maxillary first and second molars was considered as the region of interest (ROI). Bone volume fraction (BV/TV); trabecular thickness (Tb.Th); and trabecular number (Tb.N) of alveolar bone were assessed.

**Histological analysis of alveolar bone**

After micro-CT scans, samples were decalcified with 10% ethylenediaminetetraacetate (EDTA; pH 7.4) at 4℃ for 1 month, embedded in paraffin and sectioned in sagittal direction serially with a thickness of 5 μm. Hematoxylin-eosin (G1121, Solarbio, Beijing, China) was used to stain the sections in preparation for histological analysis. Image was acquired with a slide scanner (Slideview VS200, Olympus, Japan).

The paraffin sections were dewaxed, rehydrated, immersed in sodium citrate buffer for antigen retrieval, and blocked with 3% hydrogen peroxide. The sections were incubated with rabbit monoclonal IgG-anti FTO (1:200, 45980, CST, MA, USA) at 4℃ overnight, and detected with Alexa Fluor 555-labeled Donkey Anti-Rabbit IgG (1:1000, A0453, Beyotime) for 1 h at room temperature. The nuclei were stained with DAPI for 10 min. Imaging was performed using a fluorescence microscope.

**M^6^A-RNA immunoprecipitation sequence (MeRIP-seq) and data analysis**

The m^6^A modified RNAs were sequenced by MeRIP-seq at Novogene (Beijing, China) (Dominissini et al. 2013). Briefly, a total of 2 μg RNAs were extracted from the alveolar bone of normal and diabetic periodontitis mice. The integrity and concentration of RNAs were detected by Agilent 2100 Bioanalyzer and simpliNano spectrophotometer. Fragmented RNA was incubated for 2 hours at 4℃ with anti-m^6^A polyclonal antibody (Merk Millipore, MA, USA) in the immunoprecipitation experiment. Immunoprecipitated RNAs or Input was used for library construction with Ovation SoLo RNA-Seq System Core Kit (NuGEN, USA). The library preparations were sequenced on an Illumina Novaseq with paired-end read length of 150 bp according to the standard protocols. The sequencing was carried out with 3 independent biological replicates.

Raw data (raw reads) of fastq format were firstly processed using fastp (version 0.19.11). Clean data (clean reads) were obtained and used for downstream analysis. Reference genome and gene model annotation files were downloaded from genome website directly. Index of the reference genome was built using BWA (v0.7.12) and clean reads were aligned to the reference genome using BWA mem (v 0.7.12). After mapping reads to the reference genome, exomePeak R package (version 2.16.0) was used for the m^6^A peak identification in each anti-m^6^A immunoprecipitation group with the corresponding Input samples serving as a control, and q-value threshold of enrichment of 0.05 was used for all data sets. The m^6^A-enriched motifs of each group were identified by HOMER (version 4.9.1). In the peak calling result, each peak was corresponding on gene, in which the peak was located in its exon. These genes were supposed as peak related genes, and then Gene Ontology (GO) enrichment analysis was performed to identify the function enrichment results. GO enrichment analysis was implemented by the GOseq R package, in which gene length bias was corrected. GO terms with corrected P-value less than 0.05 were considered significantly enriched by peak related genes. We used KOBAS software (version 3.0) to test the statistical enrichment of peak related genes in KEGG pathways. Besides, the distribution of peak on different function regions, such as 5’-UTR, CDS, 3’-UTR was performed. Differential peak calling was performed using exomePeak R package (version 2.16.0) with parameters of P-value less than 0.05 and fold change more than 1. Using the same method, genes associated with different peaks were identified and also do GO and KEGG enrichment analysis. IGV software was used to visualize the matching of MeRIP and Input on the genome.

**Enzyme linked immunosorbent assay (ELISA)**

To detect the amount of AGEs in vivo, the blood was collected from mice in each group. The AGEs amount in serum were determined using enzyme-linked immunosorbent assay (ELISA) kits (CSB-E09414m, Cusabio, Wuhan, China) according to the manufacturer’s protocols.

**Cell proliferation assay**

The BMSCs were seeded in 96-well plates at 3×10^3^ cells per well and treated with AGEs with various concentrations. 10μL of CCK-8 reagent (C0038, Beyotime, Shanghai, China) were added into wells at corresponding time point and incubated for 1h. The absorbance was measure at 450 nm by Microplate Reader (SpectraMax iD5, Molecular Device, USA). The data was averaged from three independent experiments.

**TdT-mediated dUTP nick-end labeling (TUNEL) assay**

The BMSCs were seeded in 96-well plates and treated with AGEs at 150 μg/ml for 3 days. The cells were fixed with 4% polyformaldehyde for 30 mins, permeabilized with 0.3% TritonX-100 for 5 mins and incubated with TUNEL reagent (C1086, Beyotime) for 1 h. The nuclei were stained with 2-(4-Amidinophenyl)-6-indolecarbamidine dihydrochloride (DAPI) (C1005, Beyotime). The percentage of apoptotic cells in BMSCs is calculated.

**Alkaline phosphatase (ALP) and Alizarin red S (ARS) staining**

ALP staining was detected after 7 days of osteogenic induction. The cells were washed with PBS and fixed with 4% paraformaldehyde for 10 min. Then, cells were stained by using BCIP/NBT Alkaline Phosphatase Color Development Kit (C3206, Beyotime) according to the manufacturer's protocol. The relative ALP activity was quantified according to the protocol of alkaline phosphatase assay kit (A059-2, Jiancheng, Nanjing, China).

Calcium mineral deposition was determined after 21 days of osteogenic induction. The cells were washed with double distilled water (ddW), fixed with 4% paraformaldehyde for 15 min, and then stained with 2% Alizarin red S (A5533, Sigma, MO, USA) for 30min.

**Real-Time quantitative polymerase chain reaction (RT-qPCR)**

Total RNA was extracted from BMSCs using TRIzol reagent (15596018, Invitrogen, CA, USA). For Reverse transcription, 5ug of total RNA was performed with GoScript™ Reverse Transcription System (A5001, Promega, WI, USA). The cDNA was added to a master mix containing SYBR green (A6001, Promega) and primer mixture at a concentration of 10 μM.

Real time quantitative PCR was performed using the following cycling conditions: 95°C for 10min, 40 cycles of 15s at 95°C and 1min at 60°C, 95°C for 15s, 60°C for 15s and 95°C for 15s on Bio-Rad analysis system. Expression levels were normalized to those of endogenous β-actin and data was analyzed using the ΔΔ-Ct method. Fold change data are presented as means ± SEM. The sequence of the primers used for RT-qPCR assays are shown in **Supplementary file 1.**

**Western blot (WB)**

Briefly, cells were lysed in RIPA buffer supplemented with protease inhibitor on ice for 30 mins, and total protein was extracted and quantified using a BCA assay (P0011, Beyotime). 20 μg of total protein in each sample was separated by 8-10% SDS-PAGE gel and transferred to PVDF membranes. The membranes were blocked with 5% BSA and incubated overnight at 4℃ with the primary antibody. The antibodies used for WB analysis included mouse monoclonal anti-SOST (1:1000, AF-1589, R&D, MN, USA), anti-β-catenin (1:500, M1405-6, Huabio, Hangzhou, China); rabbit anti-Runx2 (1:500, ET1612-47, Huabio). anti-Bglap (1:500, bs-0470R, Bioss), anti-FTO (1:1000, 45980, CST, MA, USA), anti-YTHDF2 (1:1000, 71283, CST),anti-β-actin (1:1000, 8457, CST), anti GSK3β (1:500, ET1607-71, Huabio) and anti-phospho-GSK3β(1:500, ET1607-60, Huabio). Immunolabeling was detected using the ECL reagent and densitometry was performed using ImageJ.

**M^6^A dot blot assay**

The m^6^A dot blot assay was performed as previously described (Shen et al. 2017). Total RNA was extracted from BMSCs using TRIzol Reagent. Polyadenylated mRNA was purified by PolyATtract@ mRNA Isolation System III with Magnet stand (Z5300, Promega) and was quantified by NanoDrop 2000 (Thermo Fisher Scientific, USA). The poly(A)+ RNA samples were successively diluted to the concentration at 200, 100 and 50 ng/μl and were loaded to Hybond-N^+^ membrane (FFN13, Beyotime) with 2μl/dot. The nylon membrane were cross-linked by UV and blocked with 5% nonfat milk. m^6^A antibody (1:1000, 56593, CST) was used to incubated at 4℃ overnight. The membrane was detected with the ECL chemiluminescence kits after being incubated with HRP-conjugated goat IgG. Meanwhile, 2ul poly(A)+ RNA samples were dotted on the membrane and stained with 0.02% methylene blue (MB) in 0.3M sodium acetate (pH=5.2), followed by the scanning to present the total amount of input RNA.

**Immunofluorescence**

The BMSCs treated with LV-shFTO or siSOST were exposed to AGEs or not for 48h. The cells were fixed with 4% paraformaldehyde for 15mins, permeabilized with 0.1% TritonX-100 for 20 mins and blocked with 5% BSA for 1 hour. Cells were incubated with rabbit anti-FTO (1:500), mouse anti-SOST (1:200) or mouse anti-Runx2 (1:250) at 4℃ overnight, and then incubated with Alexa Fluor 555-labeled Donkey Anti-Rabbit IgG (1:1000, A0453, Beyotime), Alexa Fluor 488-labeled Goat Anti-Mouse IgG (1:1000, A0428, Beyotime) and DAPI for 1 h at room temperature. Images were acquired using the fluorescence microscope (EVOS FL Auto, Thermo Fisher Scientific, USA).

**TOPFlash luciferase assay**

The assay of Wnt activity was performed as previously described (Zhao 2014). Negative control, siSOST or LV-shFTO treated MC3T3 cells were cultured in 24-well plates respectively and transfected with 100 ng SuperTopFlash DNA plasmid (D2505, Beyotime), as well as 10 ng pRL-TK (D2760, Beyotime) as the reporter control using Lipofectamine 3000. After 6 hours, transfected cells were cultured in a medium supplemented with or without AGEs (150 μg/ml) for 48 hours. The luciferase assay was conducted using the dual luciferase assay reporter kit according to manufacturer’s protocols (RG028, Beyotime). The TOPflash luciferase activity was normalized by the Renilla luciferase activity.

**RNA immunoprecipitation (RIP) assay**

RIP-qPCR was performed as previously described (Ratnadiwakara and Änkö 2018). The BMSCs (2*10^7^/reaction) exposed to AGEs or MC3T3 cells infected with LV-shFTO were collected and lysed by complete RIP Lysis Buffer (100μl). Meanwhile, the protein A/G magnetic beads were incubated with antibody by rotation for 30 minutes at room temperature. 10μL RIP lysate of each reaction was kept as “Input”. The remaining RIP lysate of each sample was added to magnetic beads-antibody complex in RIP Immunoprecipitation Buffer and incubated with rotating at 4°C overnight. Each immunoprecipitate was incubated with proteinase K buffer at 55°C for 30 minutes to digest the protein. The immunoprecipitated RNAs were purified by phenol-chloroform-ethanol methods and synthesized to cDNA by Reverse Transcript Kits. The relative enrichment of SOST transcripts with m^6^A, FTO, YTHDF2 and IgG negative control was detected by RT-qPCR and normalized to the Input. Primers targeting m^6^A, FTO and YTHDF2 enriched regions of SOST were as follows:

M^6^A-F: GTGGAACGAAAGACCTGGGA,

M^6^A-R: GCAGGCTTTACATTTGGGTGG;

FTO/YTHDF2-F: AGGGCCAGAAATCACACTCC,

FTO/YTHDF2-R: AAGCCCAGTTTCCTCCAACG.

**Nuclear and cytoplasmic fraction**

The nucleus and cytoplasm of NC and FTO-knockdown BMSCs were separated by Nuclear and Cytoplasmic Protein Extraction Kit (Beyotime). The experiment was performed according to the instructions of manufacturer and RNase Inhibitor was added to avoid RNA degradation. The Subcellular fractions were collected and isolated total RNA using TRIzol reagent. The percentage of SOST transcripts in the control or FTO-knockdown BMSCs were determined by RT-qPCR. β-actin was served as loading control for cytosolic RNA, while U6 was used as loading control for nuclear RNA.

**RNA stability assay**

LV-shNC or LV-shFTO infected BMSCs were cultured with AGEs for 3 days. Actinomycin D (HY-17559, MCE, China) was added at a final concentration of 5μg/ml. Total RNA of each group at 0, 2, 4, 6 and 8 h was extracted using TRIzol reagent. The remaining quantities of SOST mRNA were detected by qPCR and β-actin was used for normalization (relative to 0 h). The RNA half-life (t_1/2_) was calculated using ln2/slope by Prism8 Software.

**Statistical analysis**

All quantitative data were presented as mean ± SEM based on at least 3 independent measurements. Differences between two groups were assessed with Student's t-tests and differences among three or more groups were compared with one-way ANOVA. All statistical analyses were performed using Prism 8.0 software (GraphPad, USA). The statistically significant difference was set as P < 0.05.

**Supplementary References**

Dominissini D, Moshitch-Moshkovitz S, Salmon-Divon M, Amariglio N, Rechavi G. 2013. Transcriptome-wide mapping of n(6)-methyladenosine by m(6)a-seq based on immunocapturing and massively parallel sequencing. Nature protocols. 8(1):176-189.

Ratnadiwakara M, Änkö M-L. 2018. Rna immunoprecipitation assay to determine the specificity of srsf3 binding to nanog mrna. Bio-protocol. 8(21):e3071.

Shen L, Liang Z, Yu H. 2017. Dot blot analysis of n6-methyladenosine rna modification levels. Bio-protocol. 7(1):e2095.

Zhao C. 2014. Wnt reporter activity assay. Bio-protocol. 4(14):e1183.
