# Supplementary material for: Advanced glycation end products impair bone marrow mesenchymal stem cells osteogenesis in periodontitis with diabetes via FTO-mediated N6-methyladenosine modification of sclerostin
Source: J Transl Med. 2023 Nov 4;21:781. doi: 10.1186/s12967-023-04630-5 (PMC10625275; doi:10.1186/s12967-023-04630-5)
Supplement: Supplementary file 4 — Additional file 4: The culture and characterization of BMSCs. [file 12967_2023_4630_MOESM4_ESM.docx]

**Additional file 4: The culture and characterization of BMSCs**

**Results**

**BMSCs isolation, culture and characterization**

Primary BMSCs from mice mandible exhibited a homogeneous spindle-shaped morphology and expressed surface antigens CD29, CD44 and CD105, but expressed CD45 at 1.25% (Fig. S1A,B).

Mineralized nodules and lipid droplets were observed by ARS and Oil red O staining respectively, indicating that the BMSCs had differentiation potential into osteogenic and adipogenic mesenchymal lineages (Fig. S1C,D).


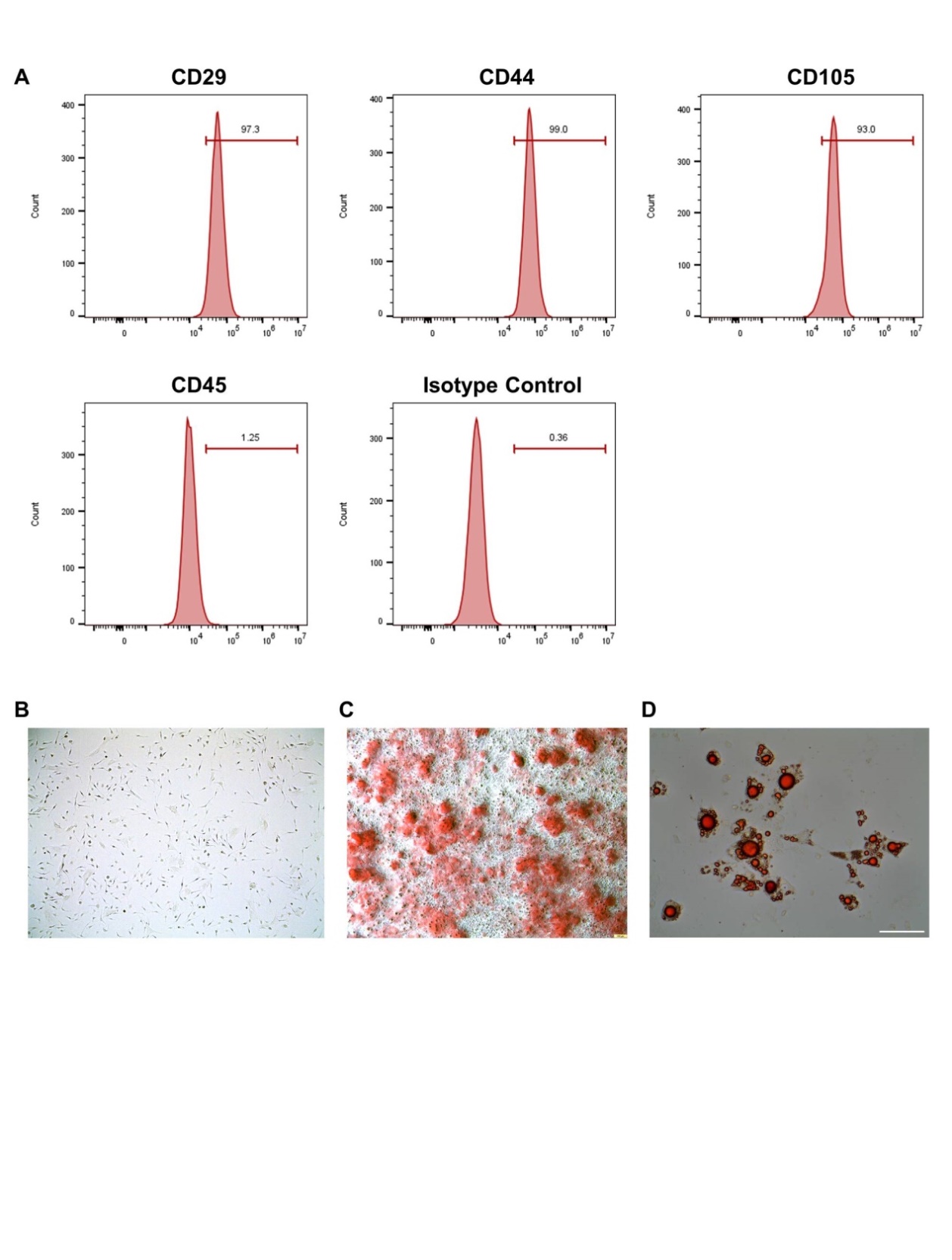


Figure S1. The culture, characterization and induced differentiation of BMSCs.

(**A**) Phenotypical characterizations of BMSCs were identified by flow cytometry.

(**B**) Morphology of BMSCs cultured for 7 days under phase-contrast micrographs.

(**C**) Mineralized extracellular matrix detected by Alizarin red S staining.

(**D**) Intracytoplasmic lipid droplets detected by oil red O staining. Scale bar = 100 µm.
