# Supplementary material for: Advanced glycation end products impair bone marrow mesenchymal stem cells osteogenesis in periodontitis with diabetes via FTO-mediated N6-methyladenosine modification of sclerostin
Source: J Transl Med. 2023 Nov 4;21:781. doi: 10.1186/s12967-023-04630-5 (PMC10625275; doi:10.1186/s12967-023-04630-5)

Additional file 5: The uncropped blots of WB

Figure S2. Uncropped blot of figure 4H

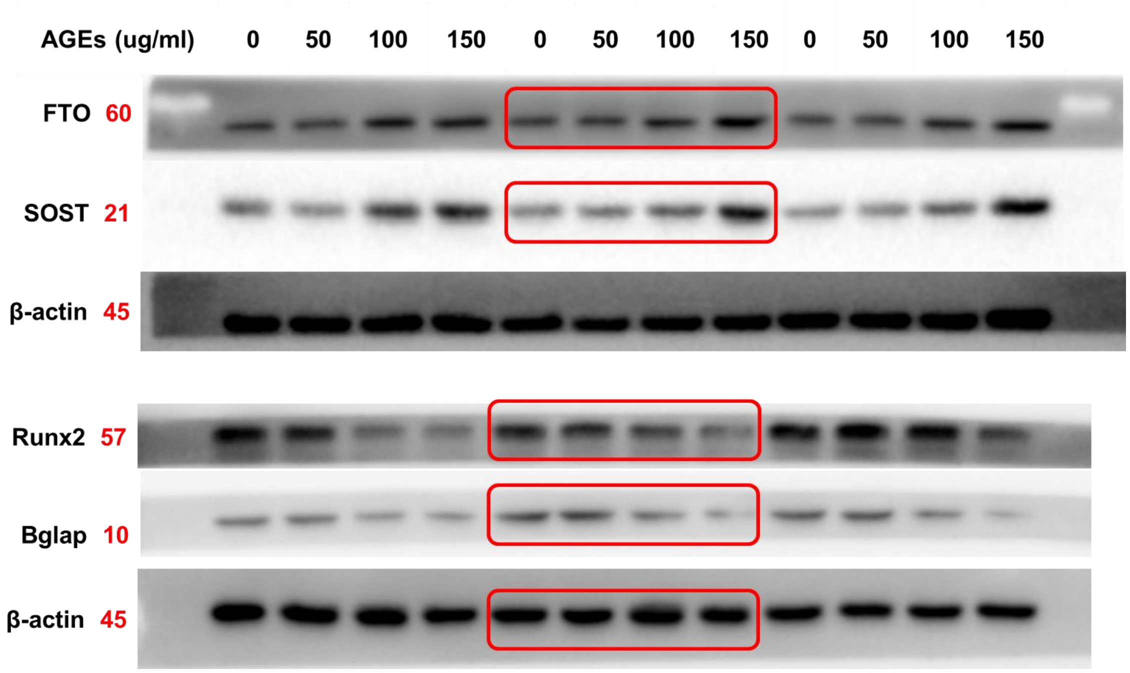

Figure S3. Uncropped blot of figure 5C

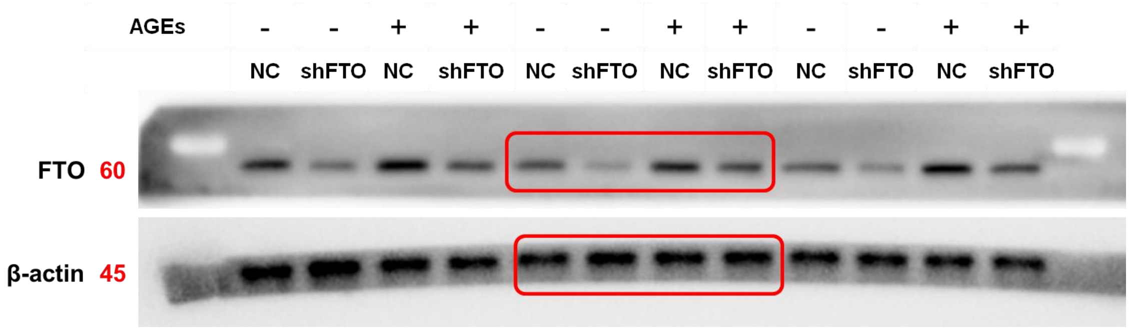

Figure S4. Uncropped blot of figure 5G

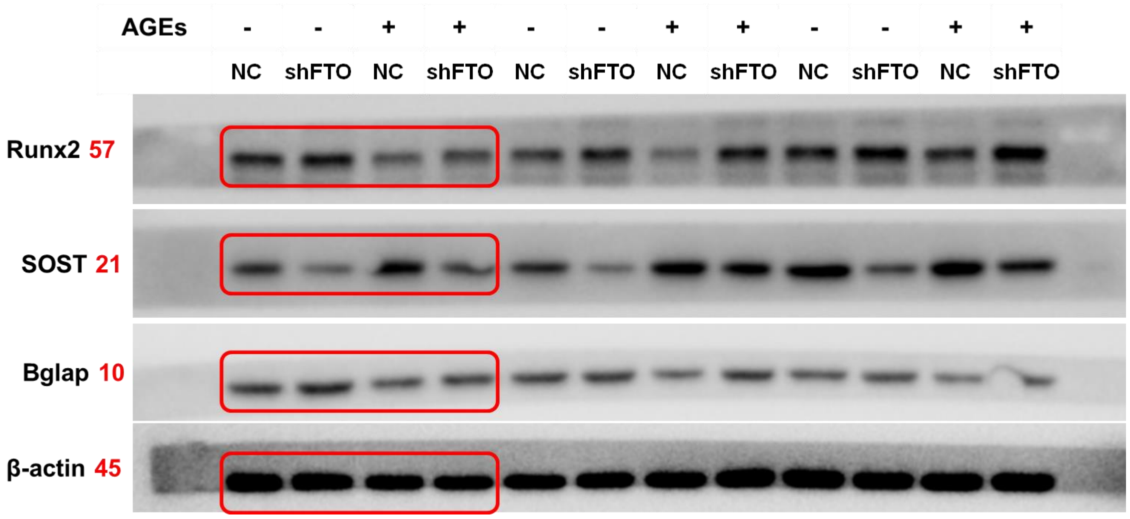

Figure S5. Uncropped blot of figure 6E

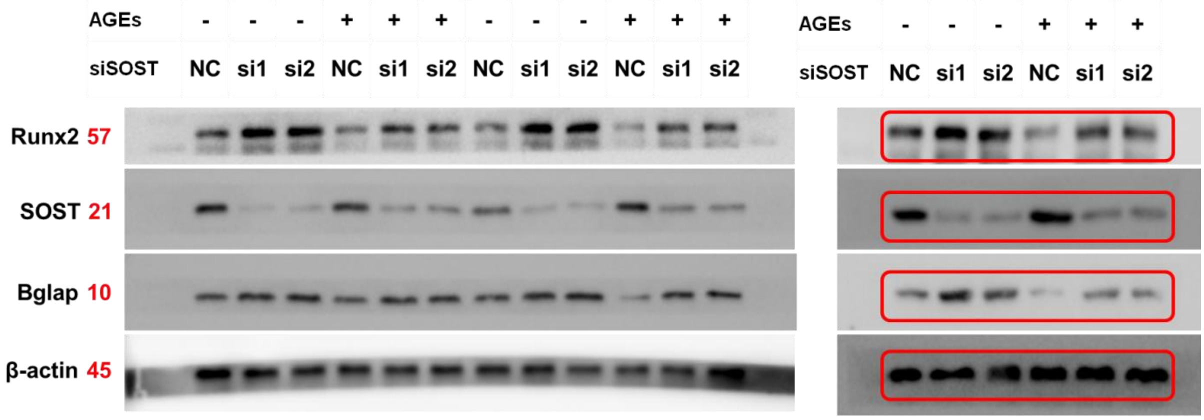

Figure S6. Uncropped blot of figure 6G

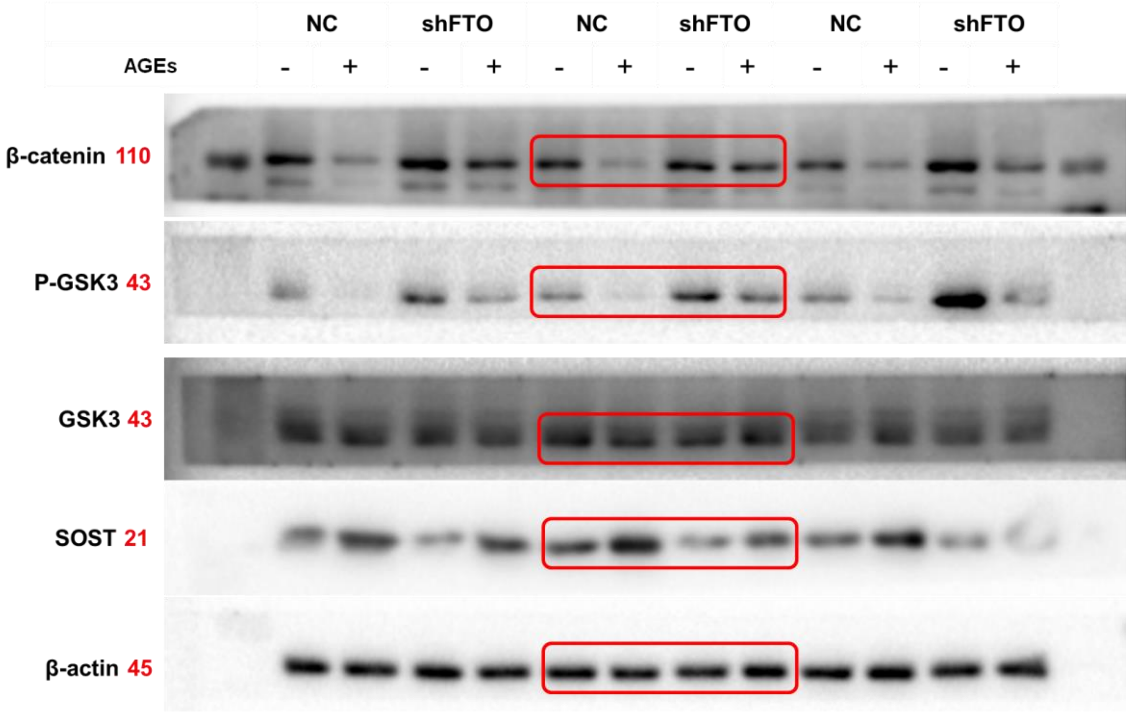

Figure S7. Uncropped blot of figure 6H

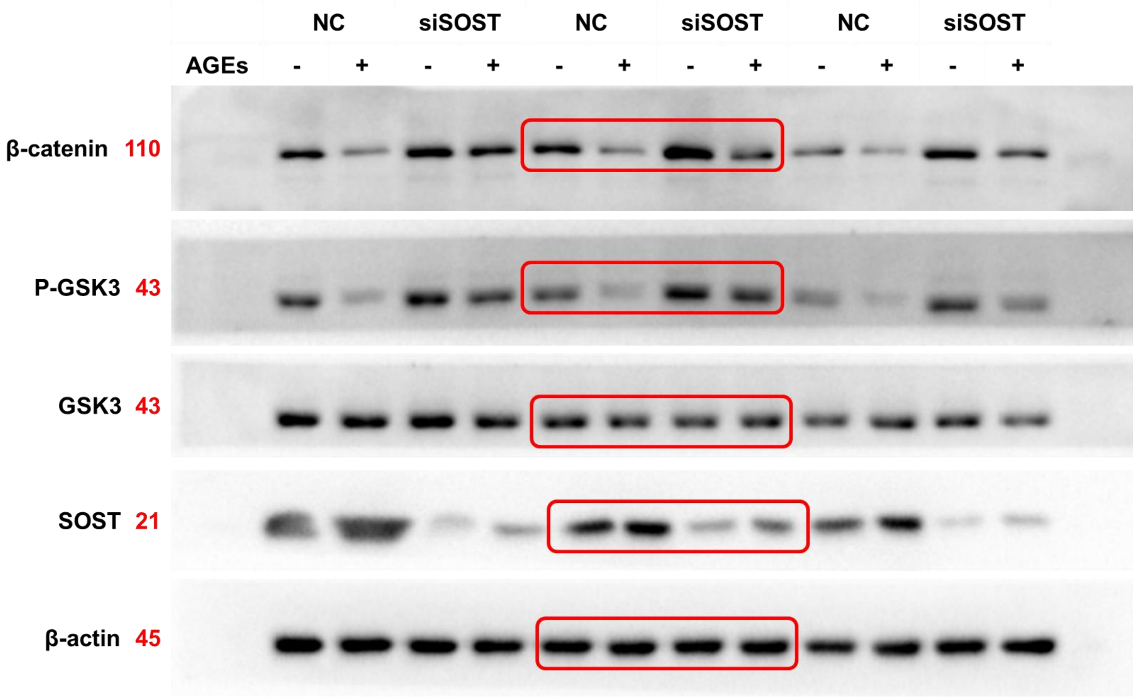

Supplement: Supplementary file 5 — Additional file 5: The uncropped blots of WB. [file 12967_2023_4630_MOESM5_ESM.pdf]
